# Beyond Estrogen: Distribution and Hormonal Correlates of Serum Testosterone Among Postmenopausal U.S. Women, NHANES 2011–2016 and 2021–2023

**DOI:** 10.3390/jcm15103607

**Published:** 2026-05-08

**Authors:** Andrew J. Goulian, Isaac Wilson, Alexander Locke

**Affiliations:** 1 College of Medicine, California Northstate University, Elk Grove, CA 95757, USA; 2 F. Edward Hébert School of Medicine, Uniformed Services University of the Health and Sciences, Bethesda, MD 20814, USA

**Keywords:** testosterone, postmenopausal women, sex hormone binding globulin (SHBG), estradiol, NHANES, hormone epidemiology, menopause

## Abstract

**Background/Objectives:** Lower circulating testosterone concentrations in postmenopausal women have been associated with adverse sexual, skeletal, and metabolic outcomes, yet population-level prevalence estimates remain inconsistent. In the absence of universally accepted diagnostic thresholds for androgen deficiency in women, interpretation of serum testosterone concentrations remains variable. This study aimed to describe the distribution of serum total testosterone and to evaluate demographic and hormonal correlates among physiologic postmenopausal women in the United States. **Methods:** This cross-sectional study analyzed women meeting criteria for physiologic menopause from the 2011–2016 and 2021–2023 National Health and Nutrition Examination Survey (NHANES) cycles. Participants using androgenic medications were excluded. Because no universally accepted diagnostic threshold exists for testosterone deficiency in women, serum total testosterone <30 ng/dL was used as an operational, population-based reference point, with <20 ng/dL evaluated as a sensitivity threshold. Survey-weighted analyses characterized the cohort and examined associations between testosterone concentrations below the <30 ng/dL operational threshold and demographic and hormonal variables using logistic regression. **Results:** Among 2707 postmenopausal women, the weighted mean total testosterone was 25.2 ± 1.1 ng/dL. Using operational, distribution-based thresholds, 56.0% of women had testosterone concentrations <20 ng/dL and 79.9% had concentrations <30 ng/dL (Rao–Scott χ^2^, *p* < 0.001). In the weighted distribution, both thresholds lay above the weighted median, with 30 ng/dL exceeding the 75th percentile. The proportion of women with testosterone concentrations below the <30 ng/dL threshold differed significantly by race/ethnicity (*p* < 0.01) and age group (*p* < 0.01), highest among Non-Hispanic Asian (87.7%) and Mexican American (89.4%) women and lowest among Non-Hispanic Black women (75.5%). In multivariable models, higher sex hormone binding globulin (SHBG) (adjusted OR = 0.720; 95% CI: 0.633–0.820; *p* < 0.001) and higher estradiol (adjusted OR = 0.577; 95% CI: 0.389–0.856; *p* < 0.05) were independently associated with lower odds of testosterone concentrations below the <30 ng/dL threshold. **Conclusions:** Testosterone concentrations below operational thresholds are highly prevalent among U.S. postmenopausal women, although estimates vary depending on the cutoff applied. Higher SHBG and estradiol levels were inversely associated with testosterone concentrations below these thresholds, underscoring the physiologic interrelationship of these hormones in postmenopausal women. These findings highlight the need for standardized, population-specific reference thresholds and clearer clinical frameworks for interpreting androgen levels in women.

## 1. Introduction

Testosterone, although traditionally regarded as a predominant male hormone, plays an essential role in the health of women across the lifespan. In postmenopausal women, androgens contribute to sexual function, bone density, muscle mass, mood regulation, and overall well-being [1]. Circulating testosterone levels in women decline gradually with age. Unlike estradiol, testosterone does not demonstrate a sharp inflection at the menopausal transition, and longitudinal data suggest that age-related decline occurs independently of menopause status [1,2]. While estrogen deficiency after menopause has long been recognized and addressed in clinical practice, androgen deficiency remains underdiagnosed, inconsistently defined, and variably treated in women [3].

Emerging evidence suggests that low testosterone in postmenopausal women is associated with adverse health outcomes, including reduced libido, fatigue, sarcopenia, osteoporosis, and unfavorable cardiometabolic profiles [4,5,6]. Despite these associations, there is no universally accepted diagnostic threshold for low testosterone in women, and laboratory reference ranges vary widely between studies and clinical laboratories [7]. Reported cutoffs therefore vary substantially across the literature, and no consensus threshold has been endorsed by regulatory or professional bodies. In this context, the present analysis applied population-based reference points (<20 ng/dL and <30 ng/dL), informed by nationally representative distribution data and baseline concentrations reported in large interventional cohorts [8,9], for descriptive comparison rather than diagnostic classification.

In addition, while testosterone therapy has been explored as a treatment for certain postmenopausal symptoms, its use remains controversial due to mixed evidence on efficacy, limited long-term safety data, and the absence of standardized prescribing guidelines from major professional societies [10]. A recent review emphasizes that the only evidence-supported indication is for hypoactive sexual desire disorder (HSDD), with no demonstrated benefit for bone, cognition, or general well-being [11].

Epidemiologic data describing the distribution and correlates of serum testosterone concentrations in postmenopausal women remain limited. The National Health and Nutrition Examination Survey (NHANES) provides a unique opportunity to address this gap by offering standardized hormonal, biochemical, demographic, and behavioral data from a representative sample of the U.S. population [12]. Understanding the distribution of serum testosterone concentrations and associated factors in postmenopausal women may help inform screening strategies, guide clinical decision-making, and shape future recommendations for assessment and management.

The present study aimed to characterize the distribution of serum testosterone concentrations in physiologic postmenopausal women using NHANES data from 2011 to 2016 and 2021 to 2023 cycles to identify demographic, hormonal, and biochemical factors associated with measured testosterone in this population. The thresholds applied (<20 ng/dL and <30 ng/dL) represent operational definitions used for population-level estimation, selected for comparability with prior NHANES-based and epidemiologic research rather than for diagnostic interpretation [8,9]. Weighted percentile positions of total testosterone were calculated within the analytic sample of postmenopausal women to contextualize applied thresholds. In the weighted distribution, both 20 ng/dL and 30 ng/dL lay above the weighted median, with 30 ng/dL exceeding the 75th percentile.

By clarifying these relationships, this study aims to contribute evidence that may support the development of consistent, evidence-based approaches to both public health policy and clinical practice in the evaluation and management of androgen status among postmenopausal women.

## 2. Materials and Methods

### 2.1. Study Design and Data Source

This cross-sectional study analyzed data from the National Health and Nutrition Examination Survey (NHANES), a nationally representative, continuous survey of the non-institutionalized U.S. population conducted by the National Center for Health Statistics (NCHS, Hyattsville, MD, USA). NHANES integrates structured interviews, standardized physical examinations, and laboratory testing to assess the health and nutritional status of adults and children in the United States [13]. Data are publicly available at https://www.cdc.gov/nchs/nhanes/ (accessed 27 April 2026).

Five NHANES cycles were analyzed, 2011–2012, 2013–2014, 2015–2016, and 2021–2023, providing a contemporary evaluation of hormonal and metabolic parameters among postmenopausal women before and after the COVID-19 pandemic. The planned 2019–2020 field cycle was suspended midway due to the pandemic; data collected during that period were subsequently combined with surplus sera from 2017 to 2018 to create the nationally representative NHANES 2017–March 2020 pre-pandemic dataset, in accordance with NCHS analytic recommendations. However, testosterone assays were not performed in this combined 2017–March 2020 cycle, and therefore data from this period were excluded from the testosterone trend analyses [14,15].

To ensure methodological comparability across survey years, serum testosterone, estradiol, and sex hormone binding globulin (SHBG) were quantified using isotope-dilution liquid chromatography–tandem mass spectrometry (ID–LC–MS/MS), which remained the reference analytic standard for sex steroid measurement throughout the included NHANES cycles. The Free Androgen Index (FAI) was calculated as (testosterone [nmol/L] ÷ sex hormone binding globulin [SHBG]) × 100, as a surrogate of bioavailable testosterone widely applied in clinical and epidemiologic studies [16]. Estradiol (E2) was included to explore estrogen–androgen interplay in the postmenopausal hormonal milieu.

Unified survey design variables and combined weights were applied across the 2011–2016 and 2021–2023 cycles following NHANES analytic guidelines [14]. This harmonization procedure accounted for differences in laboratory data availability, analytic subsampling, and behavioral covariates across cycles, preserving both population-level representativeness and cross-cycle validity for hormone-related analyses. NHANES uses a multistage probability sampling design in which counties, neighborhoods, households, and individuals are selected in successive stages to produce a nationally representative sample.

The study period was selected to capture the most recent and methodologically consistent testosterone assays available from NHANES, ensuring comparability of ID–LC–MS/MS methodology across pre- and post-pandemic cycles. A formal sample size calculation was not required, as NHANES uses a complex, multistage probability design that provides weighted estimates representative of the U.S. population, ensuring sufficient precision for national inference.

As this analysis utilized publicly available, de-identified data, it was exempt from institutional review board (IRB) oversight and conducted in accordance with the ethical standards outlined by the NCHS Research Ethics Review Board.

### 2.2. Sample Selection

Participants were drawn from the NHANES data spanning 2011–2023, encompassing cycles G (2011–2012), H (2013–2014), I (2015–2016), and L (2021–2023).

Postmenopausal status was defined using menstrual-history data available across these cycles. Women in the Reproductive Health Questionnaire dataset who both reported no menstrual periods in the past 12 months and identified “Menopause/ Change of life” as the reason, were classified as postmenopausal [17]. Women reporting hysterectomy or other causes of amenorrhea were not classified as postmenopausal. This physiologic definition was used to identify postmenopausal women across the included NHANES cycles for subsequent analytic filtering.

To minimize bias from exogenous hormone exposure, participants who reported use of testosterone or other androgenic medications in the NHANES prescription drug files were excluded. To ensure reproducibility and prevent misclassification of exogenous androgen users, the specific androgenic medications excluded in this analysis are listed. Excluded agents included testosterone (generic and brand formulations), methyltestosterone, testosterone topical gels (Androgel, Testim, Fortesta, Vogelxo), topical solution (Axiron), patches (Androderm), intranasal gel (Natesto), injectable preparations (Aveed, Xyosted, Delatestryl), testosterone pellets (Testopel), oral capsules (Jatenzo, Kyzatrex), anabolic steroids (oxandrolone, nandrolone), and the anti-androgenic diuretic spironolactone. This exclusion was operationalized by linking the NHANES prescription drug file with the drug classification file, filtering by therapeutic category codes corresponding to anabolic or androgenic agents. Participants using estrogen-containing therapies were not excluded, as the study aimed to primarily evaluate endogenous androgens in a postmenopausal cohort.

Hormone assay validity was determined using NHANES laboratory comment codes, which identify results below the lower limit of detection or otherwise invalid. Participants with missing or invalid testosterone, estradiol, or SHBG values, or with missing survey design variables were excluded.

Before sequential exclusions for androgenic medication use, missing or invalid sex-steroid measures, and incomplete covariate data, a total of 21,509 women were identified. Among these, 11,232 had available reproductive health questionnaire data, of whom 2707 met criteria for physiologic postmenopause and had complete hormone, demographic, and biochemical data available for analysis. Participants reporting androgenic medication use (*n* = 22) were excluded during the analytic filtering process prior to final cohort derivation. Missing hormone values arise primarily from NHANES laboratory subsampling rather than participant refusal, meaning that exclusions reflect survey design rather than systematic nonresponse.

**Figure 1:**
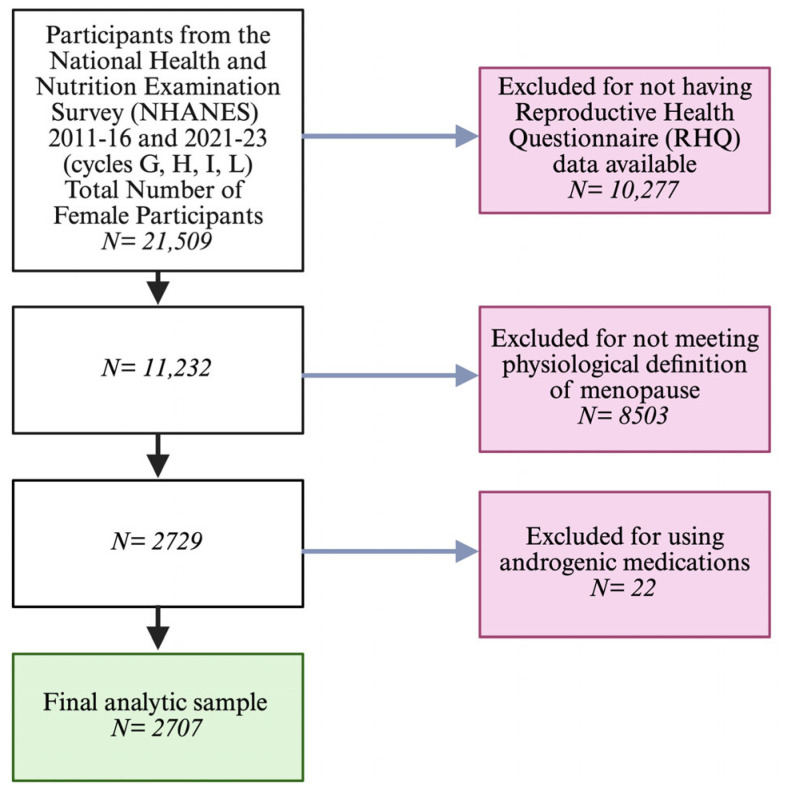
Participant selection flowchart for postmenopausal women included in NHANES 2011–2016 and 2021–2023. The analytic cohort was restricted to physiologic postmenopausal women with available serum hormone measurements, complete demographic and laboratory data, and without evidence of exogenous androgen use. The 2017–March 2020 pre-pandemic cycle was excluded because total testosterone was not assayed in that dataset.

Participants missing any covariates required for multivariable modeling were excluded using complete-case analysis to maintain the validity of NHANES weights. While this approach ensured consistency across analyses and interpretability of weighted estimates, it may limit generalizability due to reduced sample size. The participant selection process and exclusion criteria are summarized in Figure 1.

### 2.3. Outcome Variable

The primary analytic outcome was total testosterone concentration categorized relative to prespecified operational thresholds. For epidemiologic comparison, serum total testosterone <30 ng/dL was used as a distribution-based reference point. Because no universally accepted diagnostic cut-off exists for androgen deficiency in postmenopausal women, this threshold was selected to reflect the lower spectrum of testosterone concentrations observed in nationally representative data rather than a clinical definition of deficiency.

In prior NHANES analyses of U.S. females, total testosterone concentrations span a broad distribution [8]. In parallel, baseline total testosterone values in large postmenopausal interventional cohorts commonly fall below 30 ng/dL [9]. In the survey-weighted distribution of serum testosterone across the full analytic sample of postmenopausal women, operational thresholds of <20 ng/dL and <30 ng/dL were selected to represent the lower range of the observed population distribution. Accordingly, these values were used as distribution-based reference points for population description rather than diagnostic classification. Sensitivity analyses re-estimated models using the more stringent <20 ng/dL threshold to evaluate robustness across different positions within the postmenopausal testosterone distribution.

Total testosterone was quantified from fasting morning blood samples using isotope dilution–liquid chromatography–tandem mass spectrometry (ID-LC–MS/MS), as documented in the NHANES laboratory methodology [18]. NHANES laboratory documentation was reviewed to confirm methodological consistency between survey years. Although minor updates in instrumentation or laboratory procedures may have occurred between survey years, NHANES standardization and quality control protocols were applied to maintain cross-cycle consistency. NHANES laboratory comment codes were reviewed to exclude values flagged as below the limit of detection or otherwise invalid. All analyses incorporated NHANES-provided survey weights to generate nationally representative estimates accounting for the complex, multistage probability sampling design [13].

### 2.4. Predictor Variables and Statistical Analysis

Candidate predictor variables were selected a priori based on biologic plausibility and prior literature linking demographic, cardiometabolic, behavioral, and hormonal factors to testosterone levels in postmenopausal women [19,20,21]. Demographic covariates included age (years) and self-reported race/ethnicity, categorized as Mexican American, Other Hispanic, Non-Hispanic White, Non-Hispanic Black, Non-Hispanic Asian, and Other/Multi-racial. Cardiometabolic variables included body mass index (BMI, kg/m^2^), systolic blood pressure (SBP, mmHg), and diastolic blood pressure (DBP, mmHg), calculated as the mean of available readings. Behavioral predictors included average nightly sleep duration (hours) and depressive symptoms assessed by the Patient Health Questionnaire-2 (PHQ-2), scored from 0 to 6. Hormonal measures comprised serum estradiol (pg/mL) and sex hormone binding globulin (SHBG, nmol/L), both measured using standard NHANES laboratory protocols. The Free Androgen Index (FAI) was calculated as (testosterone [nmol/L] ÷ sex hormone binding globulin [SHBG, nmol/L]) × 100 to assess relative bioavailable testosterone.

Dehydroepiandrosterone sulfate (DHEAS), an adrenal androgen precursor, was not measured consistently across all included NHANES cycles and therefore was not incorporated into pooled primary models. However, a cycle-restricted sensitivity analysis including DHEAS was conducted using NHANES 2021–2023 data. Similarly, gonadotropins, follicle-stimulating hormone (FSH) and luteinizing hormone (LH) were available only in the 2021–2023 data and were evaluated in a separate cycle-restricted sensitivity analysis.

FAI was evaluated in separate complementary analyses and was not entered simultaneously with SHBG in the primary multivariable model, in order to avoid structural overadjustment.

All analyses were conducted using R version 4.5.0 (R Foundation for Statistical Computing, Vienna, Austria; https://www.r-project.org/, accessed 27 April 2026). NHANES analytic guidelines were applied for combining the 2011–2012, 2013–2014, 2015–2016, and 2021–2023 cycles, with the use of sample weights, strata, and primary sampling units to account for the complex survey design [14]. Participant characteristics were summarized as survey-weighted means with standard errors for continuous variables and as weighted frequencies and percentages for categorical variables.

To aid interpretation for readers less familiar with complex survey data, we included brief clarifications of the statistical methods used. Survey weights ensure that estimates reflect the U.S. population rather than only the sampled participants. The Rao–Scott χ^2^ test is a design-adjusted version of the standard χ^2^ test that accounts for NHANES’ multistage sampling. Similarly, survey-weighted logistic regression incorporates the sampling structure to provide nationally representative estimates of association [22].

The proportion of women with testosterone concentrations below prespecified operational thresholds was estimated using <30 ng/dL as the primary cut point and <20 ng/dL as a sensitivity cut point. Differences in the proportion of women below each operational threshold were formally tested using a survey-weighted Rao–Scott χ^2^ statistic. Weighted mean testosterone concentrations were calculated separately for each NHANES cycle, and temporal trends were assessed using survey-weighted regression models with cycle coded as an ordinal variable. Cycles were coded numerically (2011–2012 = 1, 2013–2014 = 2, 2015–2016 = 3, 2021–2023 = 4) to evaluate linear trends across survey cycles.

The proportion of women with testosterone concentrations below the operational threshold was estimated across race/ethnicity, age, and BMI categories, with subgroup differences evaluated using Rao–Scott χ^2^ tests. Survey-weighted logistic regression models were fit to estimate adjusted odds ratios (ORs) and 95% confidence intervals (CIs) for predictors of testosterone concentrations below the operational threshold. The fully adjusted model included demographic, behavioral, hormonal, and cardiometabolic covariates selected a priori.

Weighted estradiol quartiles (Q1–Q4) were derived using survey-weighted quantile estimation to account for the NHANES complex sampling design, corresponding to weighted quartile cut-points observed in the analytic sample. The proportion of women with testosterone concentrations <30 ng/dL was compared across estradiol quartiles using Rao–Scott χ^2^ tests, and these quartile groupings were subsequently incorporated into regression analyses to assess adjusted trends.

Associations were expressed as adjusted odds ratios with 95% confidence intervals. Analyses were conducted using the <30 ng/dL threshold, and a two-sided *p*-value <0.05 was considered statistically significant.

## 3. Results

### 3.1. Participant Characteristics

A total of 2707 women meeting criteria for physiologic menopause were included after exclusions for androgen-related prescriptions, invalid testosterone data, and missing covariates. The weighted mean ± SE age was 63.8 ± 0.3 years, and the weighted mean ± SE body-mass index (BMI) was 30.4 ± 0.2 kg/m^2^.

By BMI category, 39.6% of women were obese, 29.6% overweight, 29.1% normal weight, and 1.7% underweight. The weighted racial and ethnic distribution was 73.0% Non-Hispanic White, 9.0% Non-Hispanic Black, 5.1% Mexican American, 5.5% Other Hispanic, 7.5% Non-Hispanic Asian, and 7.5% Other or multiracial. These proportions reflect the restriction to physiologic menopause and exclusion of surgical causes of amenorrhea in the dataset. Weighted mean ± SE systolic and diastolic blood pressures were 128.2 ± 0.6 mmHg and 71.8 ± 0.4 mmHg, respectively. Participants reported an average weighted sleep duration of 7.6 ± 0.05 h per night.

For hormonal measures, the weighted mean ± SE total testosterone was 25.2 ± 1.07 ng/dL, estradiol 10.1 ± 0.57 pg/mL, and sex hormone binding globulin (SHBG) 64.4 ± 1.15 nmol/L, yielding a weighted mean ± SE Free Androgen Index (FAI) of 1.66 ± 0.05.

### 3.2. Comparison of Operational Testosterone Thresholds

**Figure 2:**
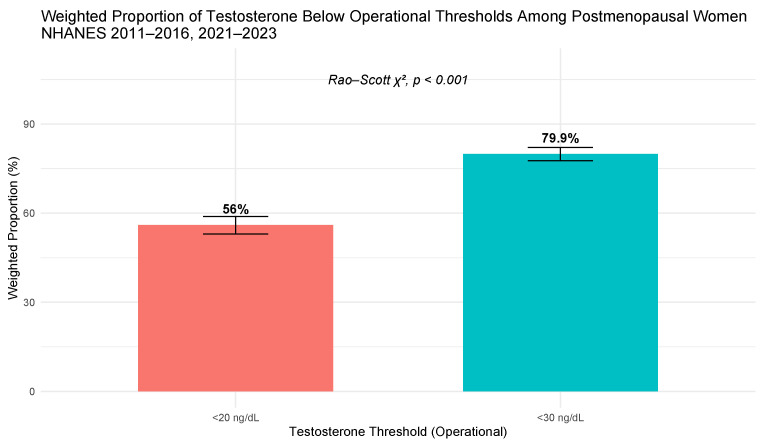
Weighted proportion of women with testosterone concentrations <30 ng/dL (operational threshold) among postmenopausal women aged ≥51 years, NHANES 2011–2016 and 2021–2023, using thresholds of <20 ng/dL and <30 ng/dL. Error bars represent 95% confidence intervals.

The proportion of postmenopausal women with testosterone concentrations below the operational thresholds varied depending on the cutoff applied (Table 1, Figure 2). Using a threshold of <20 ng/dL, 56.0% of postmenopausal women had testosterone concentrations below this level, whereas 79.9% had concentrations <30 ng/dL. The difference between thresholds was statistically significant (Rao–Scott χ^2^, F = 443.1, df = 1, *p* < 0.001).

In the survey-weighted distribution of total testosterone among postmenopausal women, the 25th, 50th, and 75th percentiles were 12.2 ng/dL, 18.3 ng/dL, and 27.4 ng/dL, respectively, indicating that both 20 ng/dL and 30 ng/dL lie above the weighted median of the analytic sample and therefore capture a majority of postmenopausal women in the distribution.

### 3.3. Temporal Trends in Mean Testosterone

In the weighted analysis of postmenopausal women from NHANES 2011–2023, the proportion of women with testosterone concentrations <30 ng/dL remained stable across cycles. The weighted proportion of women with testosterone concentrations <30 ng/dL was 80.8% (SE 1.78) in 2011–2012, 81.2% (SE 2.41) in 2013–2014, 81.2% (SE 2.41) in 2015–2016, and 77.9% (SE 1.66) in 2021–2023. This change was not statistically significant (OR = 0.95, 95% CI 0.89–1.02; *p*-trend = 0.177), suggesting no appreciable temporal shift in the proportion of postmenopausal women with testosterone concentrations below the operational threshold.

### 3.4. Demographic Associations with Operational Testosterone Thresholds

In weighted analyses, the proportion of women with testosterone concentrations <30 ng/dL differed significantly by both race/ethnicity (*p* < 0.01) and age group (*p* < 0.01). The proportion of women below the threshold was highest among Non-Hispanic Asian women (87.7%; 95% CI: 81.9–93.6) and Mexican American women (89.4%; 95% CI: 84.5–94.2), and lowest among Non-Hispanic Black women (75.5%; 95% CI: 70.3–80.7).

By age group, the proportion below the threshold ranged from 83.6% (95% CI: 79.5–87.7) among women aged 51–59 years to 73.3% (95% CI: 68.6–78.0) among those aged 70–79 years, with a slight increase to 79.9% (95% CI: 74.0–85.7) in women aged 80 years and older (Rao–Scott *p* < 0.01). This pattern suggests a modest decline in the proportion of women with testosterone concentrations below the operational threshold with advancing age.

In contrast, differences by BMI category were not statistically significant (*p* = 0.15). Weighted prevalence ranged from 79.3% (95% CI: 51.4–100.0) among underweight women to 84.0% (95% CI: 80.7–87.3) among those with normal BMI, 79.1% (95% CI: 74.7–83.5) in overweight women, and 77.6% (95% CI: 74.2–81.0) in obese women.

Collectively, these results indicate that testosterone concentrations <30 ng/dL were more prevalent among Non-Hispanic Asian and Mexican American women and declined modestly with increasing age, whereas BMI was not independently associated with the proportion of women below the threshold. Some subgroup estimates exhibited wide confidence intervals, reflecting reduced precision from smaller weighted sample sizes (Table 2).

Using the more stringent cutoff of <20 ng/dL, subgroup patterns were similar but attenuated in magnitude (Table S1). Prevalence differed significantly by race/ethnicity (Rao–Scott *p* < 0.01), ranging from 54.1% (95% CI: 50.4–57.8) among Non-Hispanic White women to 70.2% (95% CI: 63.6–76.8) among Mexican American women; Non-Hispanic Asian women also had elevated prevalence (65.2%; 95% CI: 58.8–71.5). Differences by age group and BMI category were also not statistically significant in the sensitivity analysis.

### 3.5. Multivariable Associations with Testosterone Below the <30 ng/dL Operational Threshold

In the fully adjusted survey-weighted logistic regression model using the <30 ng/dL operational cutoff (Table 3), higher sex hormone binding globulin (SHBG), higher estradiol, and older age were each independently associated with lower odds of having testosterone concentrations below this threshold. Specifically, higher SHBG was significantly associated with reduced odds of testosterone concentrations <30 ng/dL (adjusted OR = 0.720 per 1-SD increase; 95% CI: 0.633–0.820; *p* < 0.001), and higher estradiol showed a modest inverse association (adjusted OR = 0.577 per 1-SD increase; 95% CI: 0.389–0.856; *p* < 0.05). Older age was also associated with lower odds (adjusted OR = 0.816 per 1-SD increase; 95% CI: 0.692–0.964; *p* < 0.05).

Compared with Non-Hispanic White women, Mexican American (OR = 0.384; 95% CI: 0.194–0.758; *p* < 0.01), Other Hispanic (OR = 0.549; 95% CI: 0.350–0.861; *p* = 0.01), and Non-Hispanic Black (OR = 0.515; 95% CI: 0.305–0.868; *p* < 0.05) women had significantly lower odds of testosterone concentrations below the <30 ng/dL operational threshold. No significant associations were observed for BMI, blood pressure, sleep duration, or PHQ-2 depressive symptoms.

Taken together, these findings indicate that higher SHBG and estradiol levels are consistently linked to lower odds of testosterone concentrations below the operational threshold among postmenopausal women, while differences across racial and ethnic groups remain significant even after adjusting for physiologic and behavioral covariates. Multicollinearity diagnostics were assessed using generalized variance inflation factors, with all adjusted GVIF values close to 1, indicating no evidence of problematic multicollinearity (Supplementary Table S7).

### 3.6. Estradiol Quartiles and Free Androgen Index (FAI)

Weighted analyses demonstrated a graded inverse relationship between estradiol levels and the proportion of women with testosterone concentrations <30 ng/dL. As seen in Table 4, across estradiol quartiles, prevalence decreased from 88.2% (95% CI: 84.9–91.4) in the lowest quartile (Q1 < 4.21 pg/mL) to 58.2% (95% CI: 52.2–64.2) in the highest quartile (Q4 > 10.3 pg/mL) (Rao–Scott χ^2^ *p* < 0.001). This trend remained consistent after adjustment for age, BMI, race/ethnicity, and SHBG levels.

In sensitivity analyses using the stricter <20 ng/dL cutoff, the inverse gradient persisted. Weighted prevalence declined from 66.9% (95% CI: 62.3–71.5) in Q1 to 29.4% (95% CI: 24.0–34.8) in Q4 (Rao–Scott *p* < 0.001) (Table S2), supporting the robustness of the estradiol-associated pattern under alternative operational definitions.

The Free Androgen Index (FAI), calculated as (testosterone [nmol/L] ÷ SHBG [nmol/L]) × 100, demonstrated the expected strong inverse association with SHBG (*p* < 0.001), reflecting reduced bioavailable androgen levels with increasing SHBG concentrations. Beyond SHBG, FAI was significantly positively associated with BMI (β = 0.05 per kg/m^2^; *p* < 0.001) and mean diastolic blood pressure (β = 0.01 per mmHg; *p* < 0.01), and significantly inversely associated with age (β = −0.02 per year; *p* < 0.05). No statistically significant associations were observed for sleep duration (*p* = 0.71), mean systolic blood pressure (*p* = 0.30), PHQ-2 status (*p* = 0.29), or estradiol as a continuous predictor (*p* = 0.074). Survey-weighted mean FAI also differed significantly by race/ethnicity (*p* = 0.003), BMI category (*p* < 0.001), and age group (*p* < 0.001) (Table S3a,b).

### 3.7. Hormonal Sensitivity Analysis (DHEAS, FSH, and LH), NHANES 2021–2023

In cycle-restricted sensitivity analyses limited to NHANES 2021–2023 participants, adrenal and pituitary hormones were incorporated into survey-weighted logistic regression models evaluating associations with testosterone <30 ng/dL using model-specific complete-case samples.

In the first sensitivity model including DHEAS (Table S4), the weighted mean DHEAS concentration was 1.36 µmol/L (SE 0.05). Higher DHEAS concentrations were significantly associated with lower odds of testosterone <30 ng/dL (adjusted OR 0.55; 95% CI 0.47–0.63; *p* < 0.001). In this cycle-restricted model, SHBG remained significantly inversely associated with testosterone <30 ng/dL (adjusted OR 0.99; 95% CI 0.98–0.99; *p* < 0.001), while estradiol was no longer statistically significant. Older age, lower BMI, and greater sleep duration were also associated with lower odds of testosterone <30 ng/dL, and Other Hispanic ethnicity remained associated with lower odds relative to the reference group.

A second sensitivity model simultaneously incorporating DHEAS, FSH, and LH was then estimated (Table S5). The weighted mean FSH concentration was 67.43 mIU/mL (SE 1.14) and the weighted mean LH concentration was 34.09 mIU/mL (SE 0.46). In adjusted analyses, LH was significantly inversely associated with testosterone <30 ng/dL (adjusted OR 0.96; 95% CI 0.94–0.99; *p* = 0.006), whereas FSH demonstrated a non-significant positive trend (adjusted OR 1.01; 95% CI 1.00–1.03; *p* = 0.086).

In this cycle-restricted model, DHEAS, SHBG, age, BMI, and greater sleep duration remained significantly associated with the outcome, and Other Hispanic ethnicity was associated with lower odds of testosterone <30 ng/dL relative to the reference group. Most other race/ethnicity categories were not statistically significant, and estradiol and FSH were not independently associated with the outcome in this model.

## 4. Discussion

### 4.1. Summary of Key Findings

This discussion contextualizes the observed hormonal patterns within postmenopausal physiology and highlights their implications for interpreting testosterone in clinical gynecologic endocrinology. In this nationally representative study of 2707 women meeting criteria for physiologic menopause from NHANES 2011–2016 and 2021–2023, nearly four of five participants had total testosterone concentrations below the <30 ng/dL operational threshold. Weighted analyses demonstrated that the proportion of women below the operational threshold was highly sensitive to the cutoff applied: 56.0% of women had concentrations <20 ng/dL compared with 79.9% below <30 ng/dL, a statistically significant difference (Rao–Scott χ^2^, *p* < 0.001).

Across survey cycles, no temporal trend was observed in mean testosterone or in the proportion of women below the operational threshold, suggesting stable postmenopausal androgen distributions in the U.S. population over the past decade.

Demographically, the proportion of women with testosterone concentrations below <30 ng/dL differed significantly by race/ethnicity (*p* < 0.01) and age group (*p* < 0.01). The proportion of women below the operational threshold was highest among Non-Hispanic Asian (87.7%) and Mexican American (89.4%) women and lowest among Non-Hispanic Black (75.5%) women. The proportion below the threshold declined modestly with advancing age, from 83.6% among women aged 51–59 years to 73.3% among those aged 70–79 years, while BMI was not significantly associated with the operational definition.

In fully adjusted, survey-weighted logistic regression models, higher sex hormone binding globulin (SHBG) and higher estradiol concentrations were independently associated with lower odds of having testosterone concentrations below the <30 ng/dL operational threshold, and older age was similarly inversely associated. Specifically, SHBG (OR = 0.720 per 1-SD increase; 95% CI 0.633–0.820; *p* < 0.001), estradiol (OR = 0.577 per 1-SD increase; 95% CI 0.389–0.856; *p* < 0.05), and older age (OR = 0.816 per 1-SD increase; 95% CI 0.692–0.964; *p* < 0.05) were each independently associated with lower odds of meeting the <30 ng/dL operational threshold. After adjustment, Mexican American, Other Hispanic, and Non-Hispanic Black women had significantly lower odds of concentrations below the threshold compared with Non-Hispanic White women (*p* < 0.05). No independent associations were observed for BMI, blood pressure, sleep duration, or depressive symptoms.

Further analyses stratified by estradiol quartile confirmed a graded inverse relationship between estradiol and the proportion of women below the <30 ng/dL threshold (*p* < 0.001), decreasing from 88.2% in the lowest quartile (<4.21 pg/mL) to 58.2% in the highest quartile (>10.3 pg/mL). The Free Androgen Index (FAI) demonstrated expected correlations, with higher SHBG strongly associated with lower FAI and lower odds of concentrations below the operational threshold (*p* < 0.001). Collectively, these findings underscore the interdependence of estradiol, SHBG, and total testosterone in the postmenopausal hormonal milieu and highlight persistent racial and ethnic variation in androgen profiles among U.S. women.

### 4.2. Comparison with Prior Literature

Our prevalence estimates align with prior epidemiologic studies reporting that the majority of postmenopausal women exhibit total testosterone concentrations within the lower range of the postmenopausal distribution [21]. These findings also parallel longitudinal observations from the Study of Women’s Health Across the Nation (SWAN), which demonstrated significant racial and ethnic variation in sex steroid concentrations across the menopausal transition, independent of age and body mass index [23]. In that multiethnic cohort, circulating estradiol and androgen levels varied systematically by race/ethnicity and adiposity, underscoring the importance of population-level context when interpreting hormonal values in midlife and older women. The consistency between SWAN and the present nationally representative NHANES analysis suggests that observed racial differences in androgen profiles likely reflect physiologic variation rather than pathologic deviation.

The inverse association between sex hormone binding globulin (SHBG) and testosterone concentrations below the operational threshold observed in this study is consistent with established endocrine physiology, whereby higher SHBG concentrations increase androgen-binding capacity and reduce the proportion of bioavailable testosterone [24].

Similarly, the inverse association between estradiol and testosterone concentrations below the threshold aligns with established steroidogenic interrelationships in postmenopausal women. Peripheral aromatization of adrenal and ovarian androgen precursors contributes to circulating estradiol production, reflecting shared upstream steroidogenic pathways [25]. Together, these findings reinforce the physiologic coupling of estradiol, SHBG, and testosterone in the postmenopausal hormonal milieu.

In sensitivity analyses restricted to NHANES 2021–2023, adjustment for DHEAS did not materially modify the associations observed in the primary pooled models. Higher DHEAS concentrations were independently associated with lower odds of testosterone <30 ng/dL. Importantly, inclusion of this adrenal androgen measure did not substantively change the relationships between testosterone, SHBG, estradiol, and demographic factors, supporting the robustness of the primary findings.

In additional cycle-restricted analyses incorporating gonadotropins, FSH and LH, inclusion of these pituitary markers did not materially alter the overall pattern of associations observed in pooled models. LH demonstrated an inverse association with testosterone <30 ng/dL, whereas FSH showed a non-significant positive trend. DHEAS, SHBG, age, BMI, sleep duration, and Other Hispanic ethnicity remained associated with the outcome in this cycle-restricted model. Although the cross-sectional design precludes causal inference, these supplemental analyses support the stability of the primary findings across broader components of the endocrine axis.

The modest decline in the proportion of women with testosterone concentrations below the operational threshold with advancing age warrants consideration. Age-related increases in SHBG concentrations have been described in postmenopausal populations and may influence the distribution of circulating total testosterone values [26]. Additionally, rising LH levels after menopause, reflecting reduced estrogen-mediated negative feedback, may contribute to persistent androgen production and could partially explain this age-related pattern. Postmenopausal ovaries retain limited steroidogenic capacity, and increased LH during the menopausal transition has been associated with enhanced androgen production through stimulation of steroidogenic pathways [27]. Moreover, longitudinal studies of the menopausal transition demonstrate relative stabilization of sex steroid concentrations following menopause [28]. A healthy survivor effect may also contribute, whereby older women in nationally representative samples represent a metabolically healthier subgroup [29]. Given the cross-sectional design, these findings should not be interpreted as evidence of longitudinal hormonal increases with age, but rather as reflecting population-level distributional patterns across older age strata.

### 4.3. Biological Mechanisms and Clinical Implications

A central finding of this analysis is the large proportion of postmenopausal women with total testosterone concentrations below the <30 ng/dL operational threshold. Because this threshold lies above the weighted median and exceeds the 75th percentile of the postmenopausal testosterone distribution, these findings suggest that values within this range may reflect typical menopausal physiology rather than universal androgen insufficiency. When a substantial proportion of a population falls below a given reference point, careful contextual interpretation is warranted, particularly in the absence of accompanying clinical criteria. Accordingly, testosterone concentrations in this range likely reflect the expected postmenopausal hormonal milieu rather than unequivocal evidence of pathologic deficiency. These findings underscore the importance of interpreting testosterone levels within age- and sex-specific physiologic context rather than extrapolating thresholds derived from premenopausal or mixed populations. In this setting, lower testosterone concentrations are consistent with the baseline endocrine state of menopause in the absence of ovarian androgen production [30,31].

This finding underscores the urgent need for context-specific reference ranges, since cutoffs derived from premenopausal or mixed populations risk overestimating biochemical deficiency and misclassifying aging physiology as disease. As shown in the weighted distribution of the analytic sample, the applied thresholds correspond to positions in the upper portion of the observed postmenopausal testosterone distribution. Appropriately, the proportion of women below these operational thresholds should be interpreted as a population-based descriptor of the hormone distribution rather than a diagnostic classification.

Against this backdrop, the inverse associations observed with SHBG and estradiol remain physiologically coherent. Higher SHBG was consistently associated with lower odds of testosterone concentrations below the operational threshold, reflecting its regulatory role in androgen transport and bioavailability [32]. Likewise, higher estradiol was linked to lower odds of concentrations below the threshold, consistent with shared steroidogenic pathways in which androgen precursors are aromatized to estrogens and the two hormones rise in tandem [25]. However, interpretation of the SHBG association warrants caution. Because SHBG directly binds circulating testosterone and influences measured total testosterone concentrations, part of the observed relationship may reflect biochemical interdependence between SHBG and total testosterone measurements rather than complete physiologic independence. Estradiol also increases hepatic SHBG synthesis, further reinforcing the interconnected nature of these hormonal pathways. Although NHANES does not provide complete information on exogenous estrogen therapy across all included cycles, the SHBG–testosterone pattern observed in this analysis is consistent with expected physiologic variation in postmenopausal women.

In cycle-restricted sensitivity analyses, higher DHEAS concentrations were independently associated with lower odds of testosterone concentrations below the operational threshold. This observation is consistent with prior literature demonstrating that DHEAS serves as a major adrenal precursor for peripheral androgen synthesis in postmenopausal women [33]. Although circulating DHEAS concentrations decline gradually with age, adrenal-derived androgens remain an important substrate for downstream androgen production through peripheral conversion pathways [34]. These findings support the biologic plausibility of the observed association and suggest that variation in adrenal androgen availability may partially influence the distribution of total testosterone concentrations in postmenopausal populations.

Interestingly, although obesity is typically associated with lower SHBG levels and increased peripheral aromatization of androgens to estrogens, the proportion of women with testosterone concentrations below the operational threshold was numerically lower among obese women than among those of normal weight. This finding may reflect altered androgen dynamics in obesity. Increased adiposity is associated with enhanced peripheral conversion of adrenal androgen precursors, which may contribute to the maintenance of circulating total testosterone levels despite reduced SHBG concentrations [35]. In addition, lower SHBG in obesity may alter the balance between bound and unbound hormone fractions in ways that do not uniformly reduce total testosterone concentrations. Residual confounding by age, metabolic comorbidities, or medication use may also contribute to this pattern. These findings underscore the complexity of androgen physiology in obesity and suggest that free or bioavailable testosterone measures may provide complementary insight beyond total testosterone alone.

From a clinical perspective, these results highlight that isolated total testosterone values should not be interpreted as definitive indicators of androgen insufficiency in postmenopausal women, particularly when commonly used cut points classify most women below reference thresholds. Contextual markers such as SHBG, estradiol, and potentially elements of mineral metabolism are critical for accurate interpretation, while the Free Androgen Index may provide a more informative surrogate of bioavailable androgen status when SHBG concentrations vary substantially. Accordingly, the population percentiles presented here are best viewed as descriptive epidemiologic reference points rather than stand-alone clinical decision thresholds, helping to avoid misclassifying age-related endocrine physiology as biochemical deficiency in the absence of clinical criteria. Moreover, this framework may help explain why clinical trials of testosterone therapy in postmenopausal women often enroll participants labeled as “deficient” on biochemical grounds yet demonstrate only modest or inconsistent clinical benefits [25,36]. The challenge, therefore, may lie less in biology itself than in the inappropriate application of reference values derived from dissimilar populations.

### 4.4. Limitations

Several limitations should be acknowledged when interpreting these findings. First, the cross-sectional design of NHANES precludes any inference of causality between the examined predictors and low testosterone status [37]. Second, behavioral variables such as sleep duration and depressive symptoms were self-reported and therefore subject to potential recall and reporting bias [38]. Third, although the inclusion of the 2021–2023 NHANES cycle provided a more contemporary population estimate, changes in assay methodology and the availability of hormonal measures across survey years may limit comparability and generalizability [14].

Residual confounding by unmeasured variables also remains possible, including the potential influence of menopausal hormone therapy, surgical menopause, and chronic disease burden despite multivariable adjustment [39]. Menopausal status was defined using physiologic criteria derived from reproductive health questionnaire data rather than chronological age, improving specificity compared with age-based definitions but still allowing for potential misclassification due to individual variability in hormonal transition and menstrual history [40].

Furthermore, exogenous hormone exposure may influence circulating sex steroid concentrations. Oral estrogen therapy increases SHBG levels and may lower calculated free androgen indices, while chronic glucocorticoid exposure can suppress adrenal androgen production. Although hormone therapy–related bleeding was excluded in menopausal classification, medication use was not explicitly adjusted for in the primary models and may contribute to residual confounding.

Finally, this analysis was limited to total testosterone concentrations, without direct measurement of free or bioavailable testosterone, which may provide a more physiologically relevant indicator of androgenic activity in women [24]. Given the observed influence of sex hormone binding globulin and estradiol, future studies incorporating standardized free hormone indices and harmonized assays across NHANES cycles would enhance comparability and mechanistic understanding. Complete-case exclusions were driven primarily by NHANES laboratory subsampling structure and missing covariate availability, which may reduce precision and external generalizability while being less likely to introduce systematic participant-level nonresponse bias.

### 4.5. Future Directions

Building on longitudinal evidence describing complex relationships between sex hormones and midlife health outcomes, future studies should further examine causal pathways linking testosterone concentrations, hormonal markers, and clinical symptoms in postmenopausal women [41]. In particular, prospective cohort studies could help determine whether testosterone concentrations at the lower end of the postmenopausal distribution contribute directly to adverse outcomes or instead reflect broader hormonal aging processes. Additional research should clarify the biological mechanisms underlying these associations and assess whether alterations in mineral metabolism influence androgen regulation in aging women [42].

Future work could also evaluate modifiable factors that affect SHBG, such as body composition, diet, and insulin sensitivity, to identify strategies for maintaining physiologic androgen balance [43]. In the United States, no testosterone formulation is currently approved by the Food and Drug Administration (FDA) specifically for use in women, complicating standardized treatment and dosing approaches. Regulatory and clinical research efforts need to aim to define evidence-based reference ranges and context-specific interpretive thresholds appropriate for postmenopausal physiology, distinguishing normal hormonal aging from clinically meaningful androgen insufficiency.

## 5. Conclusions

This nationally representative analysis demonstrates that when empirically derived, population-based thresholds are applied, total testosterone concentrations below commonly used cut points are highly prevalent among postmenopausal women in the United States. These concentrations likely reflect typical postmenopausal physiology rather than evidence of widespread androgen deficiency. The strong inverse associations observed with SHBG and estradiol underscore the complex hormonal interrelationships that regulate androgen balance in later life.

Although lower testosterone concentrations in women have been associated with reduced bone density, impaired sexual function, and diminished quality of life [44,45], the absence of validated, sex-specific diagnostic thresholds and the lack of U.S. Food and Drug Administration (FDA)–approved testosterone formulations for women continue to limit clinical translation [46,47]. Accordingly, the present findings should be interpreted as descriptive rather than prescriptive, reflecting population-level hormonal distributions rather than diagnostic classification.

These findings carry direct clinical relevance for gynecologic endocrinology. Commonly used cut points for testosterone categorize the majority of postmenopausal women as falling below reference thresholds, suggesting that such values may lack clinical specificity in this population. Rather than indicating pathology, these concentrations appear to represent the expected hormonal milieu of menopause. The observed relationships among testosterone, estradiol, and SHBG support a physiology-based interpretive framework in which androgen measures are evaluated relative to estrogen status, binding proteins, and postmenopausal endocrine context. By clarifying how testosterone values can be contextualized in midlife and older women, this work supports more biologically coherent interpretation in menopause care.

Future research should prioritize longitudinal and interventional studies to define causal mechanisms, assess therapeutic efficacy, and establish standardized, sex-specific, and age-adjusted reference ranges. Developing validated interpretive frameworks will be essential for distinguishing physiologic hormonal aging from clinically meaningful androgen insufficiency and for guiding evidence-based, patient-centered care in postmenopausal women.

## Figures and Tables

**Table 1:** Weighted proportion of women with testosterone concentrations below operational thresholds among postmenopausal women, NHANES 2011–2016 and 2021–2023.

Operational Threshold (ng/dL)	Weighted Prevalence (%)	SE (%)	95% CI
<20 ng/dL	56.0	1.6	52.9–59.0
<30 ng/dL	79.9	1.2	77.5–82.1

**Table 2:** Weighted Prevalence of Testosterone Below the <30 ng/dL Operational Threshold Among Postmenopausal Women, NHANES 2011–2016 and 2021–2023. Note: *p*-values from Rao–Scott χ^2^ tests for association between characteristic and testosterone concentrations below the operational threshold.

Characteristic	Weighted Prevalence (%)	95% CI	SE (%)	*p*-Value
**Race/Ethnicity**				<0.01
Non-Hispanic White	79.7	76.9–82.5	1.4	
Mexican American	89.4	84.5–94.2	2.5	
Other Hispanic	76.8	70.0–83.7	3.5	
Non-Hispanic Black	75.5	70.3–80.7	2.7	
Non-Hispanic Asian	87.7	81.9–93.6	3.0	
Other/Multi-Racial	75.4	58.8–92.0	8.5	
**Age Group (years)**				<0.01
51–59	83.6	79.5–87.7	2.1	
60–69	80.4	77.6–83.2	1.4	
70–79	73.3	68.6–78.0	2.4	
80+	79.9	74.0–85.7	3.0	
**BMI Category**				0.15
Underweight (<18.5)	79.9	51.4–100.0	14.3	
Normal (18.5–24.9)	84.0	80.7–87.3	1.7	
Overweight (25–29.9)	79.1	74.7–83.5	2.3	
Obese (≥30)	77.6	74.2–81.0	1.7	

**Table 3:** Fully adjusted survey-weighted logistic regression of testosterone <30 ng/dL (operational threshold) among postmenopausal women, NHANES 2011–2016 and 2021–2023. Odds ratios (ORs) for continuous variables are per 1-SD increase. Reference categories: race/ethnicity = Non-Hispanic White; PHQ-2 < 3.

Variable	Adjusted OR	95% CI	*p*-Value
*Race/Ethnicity*			
Mexican American	0.382	0.194–0.758	<0.01
Other Hispanic	0.549	0.350–0.861	<0.05
Non-Hispanic Black	0.515	0.305–0.868	<0.05
Non-Hispanic Asian	0.592	0.338–1.040	0.075
*Blood Pressure (per SD)*			
Mean Systolic BP	1.030	0.878–1.210	0.713
Mean Diastolic BP	0.916	0.782–1.070	0.282
BMI (per SD)	0.972	0.824–1.150	0.733
Age (per SD)	0.816	0.692–0.964	<0.05
Sleep (hours/night, per SD)	0.984	0.841–1.150	0.841
PHQ-2 ≥ 3 (yes vs. no)	0.829	0.541–1.270	0.395
Estradiol (per SD)	0.577	0.389–0.856	<0.05
SHBG (per SD)	0.720	0.633–0.820	<0.001

Note: OR = odds ratio; CI = confidence interval; BP = blood pressure; SHBG = sex hormone binding globulin; PHQ-2 = Patient Health Questionnaire-2. Continuous variables are scaled per 1-SD increase; corresponding SD magnitudes are provided in Supplementary Table S6.

**Table 4:** Weighted prevalence of testosterone below the <30 ng/dL operational threshold by estradiol quartile among postmenopausal women, NHANES 2011–2016 and 2021–2023. Estimates are weighted using the NHANES complex survey design. Quartile cut-points are based on weighted estradiol values.

Estradiol Quartile (pg/mL)	Weighted Prevalence (%)	95% CI	SE (%)	*p*-Value
Q1 (<4.21)	88.2	84.9–91.4	1.7	<0.001
Q2 (4.21–6.47)	84.9	80.5–89.2	2.2	<0.001
Q3 (6.47–10.3)	73.1	68.3–77.8	2.4	<0.001
Q4 (>10.3)	58.2	52.2–64.2	3.1	<0.001

## Data Availability

All data used in this study are publicly available from the NHANES website, maintained by the Centers for Disease Control and Prevention (CDC), at: https://www.cdc.gov/nchs/nhanes/ (accessed 27 April 2026).

## Supplementary Materials

The following supporting information can be downloaded at: https://www.mdpi.com/article/10.3390/jcm15103607/s1, Table S1. Weighted Proportion of Women with Total Testosterone <20 ng/dL Among Postmenopausal Women, NHANES 2011–2016 & 2021–2023. Table S2. Weighted prevalence of testosterone below the <20 ng/dL operational threshold by estradiol quartile among postmenopausal women, NHANES 2011–2016 & 2021–2023. Table S3a. Survey-weighted linear regression analyses examining associations between the Free Androgen Index (FAI) and clinical, demographic, and hormonal predictors among postmenopausal women, NHANES 2011–2016 & 2021–2023. Table S3b. Survey-weighted mean Free Androgen Index (FAI) by demographic and clinical characteristics among postmenopausal women, NHANES 2011–2016 & 2021–2023. Table S4. DHEAS Sensitivity Analysis (NHANES 2021–2023). Table S5. Gonadotropin Sensitivity Analysis (NHANES 2021–2023). Table S6. Standard Deviation Magnitudes for Continuous Predictors. Standard deviations of continuous variables included in the survey-weighted logistic regression models (NHANES 2011–2016 and 2021–2023). Table S7. Multicollinearity Diagnostics for the Multivariable Model. Generalized variance inflation factors (GVIF) for predictors included in the SD-scaled survey-weighted logistic regression model.
