# Supplementary material for: Beyond Estrogen: Distribution and Hormonal Correlates of Serum Testosterone Among Postmenopausal U.S. Women, NHANES 2011–2016 and 2021–2023
Source: J Clin Med. 2026 May 8;15(10):3607. doi: 10.3390/jcm15103607 (PMC13207169; doi:10.3390/jcm15103607)
Supplement: Supplementary file 1 [file jcm-15-03607-s001.zip › jcm-4142543-supplementary.pdf]

Table S1. Weighted Proportion of Women with Total Testosterone <20 ng/dL Among Postmenopausal Women, NHANES 2011–2016 & 2021–2023.

Characteristic	Weighted Proportion (%)	95% CI	SE (%)	p-Value
**Race/Ethnicity**				<0.01
Non-Hispanic White	54.1	50.4 – 57.8	1.9	
Mexican American	70.2	63.6 – 76.8	3.4	
Other Hispanic	58.5	53.2 – 63.7	2.7	
Non-Hispanic Black	55.7	49.8 – 61.7	3.0	
Non-Hispanic Asian	65.2	58.8 – 71.5	3.2	
Other/Multi-Racial	60.3	47.5 – 73.1	6.5	
**Age Group (years)**				0.296
51–59	59.4	54.1 – 64.7	2.7	
60–69	54.9	50.9 – 59.0	2.1	
70–79	53.3	47.7 – 58.8	2.8	
80+	57.0	50.0 – 64.1	3.6	
**BMI Category**				0.084
Underweight (<18.5)	63.6	41.2 – 85.9	11.4	
Normal (18.5–24.9)	60.9	56.0 – 65.7	2.5	
Overweight (25–29.9)	54.1	49.8 – 58.4	2.2	
Obese (≥30)	53.3	49.1 – 57.6	2.2	

Note: p-values from Rao–Scott χ^2 tests for association between characteristic and testosterone concentrations below the operational threshold.

Table S2. Weighted prevalence of testosterone below the <20 ng/dL operational threshold by estradiol quartile among postmenopausal women, NHANES 2011–2016 & 2021–2023.

Estradiol Quartile (pg/mL)	Weighted Prevalence (%)	95% CI	SE (%)	p-Value
Q1 (<4.21)	66.9	62.3 – 71.5	2.34	<0.001
Q2 (4.21–6.47)	56.6	51.5 – 61.8	2.63	<0.001
Q3 (6.47–10.3)	43.9	38.8 – 49.1	2.64	<0.001
Q4 (>10.3)	29.4	24.0 – 34.8	2.76	<0.001

Estimates are weighted using the NHANES complex survey design. Quartile cut-points are based on weighted estradiol values.

Table S3a. Survey-weighted linear regression analyses examining associations between the Free Androgen Index (FAI) and clinical, demographic, and hormonal predictors among postmenopausal women, NHANES 2011–2016 & 2021–2023.

Predictor	β (per unit)	95% CI	p-Value
SHBG (nmol/L)	-0.01	-0.02 to -0.01	<0.001
BMI (kg/m^2)	0.05	0.03 to 0.06	<0.001
Sleep (hours/night)	0.01	-0.03 to 0.05	0.71
Age (years)	-0.02	-0.03 to 0.00	0.013
Mean systolic BP (mmHg)	0.00	-0.01 to 0.00	0.30
Mean diastolic BP (mmHg)	0.01	0.00 to 0.02	0.002
PHQ-2 ≥3 (yes vs no)	0.32	-0.28 to 0.92	0.29

Estradiol (pg/mL)	0.02	0.00 to 0.04	0.074

Beta coefficients represent change in FAI per one-unit increase in predictor. Estimates account for the NHANES complex survey design.

Table S3b. Survey-weighted mean Free Androgen Index (FAI) by demographic and clinical characteristics among postmenopausal women, NHANES 2011–2016 & 2021–2023.

Characteristic	Weighted Mean FAI	95% CI	SE	p-Value
**Race/Ethnicity**				<0.05
Non-Hispanic White	1.41	1.31 – 1.52	0.05	
Non-Hispanic Black	1.47	1.32 – 1.62	0.08	
Non-Hispanic Asian	1.31	1.12 – 1.51	0.10	
Mexican American	1.24	1.10 – 1.39	0.07	
Other Hispanic	2.07	1.58 – 2.56	0.25	
Other/Multi-Racial	2.16	0.79 – 3.52	0.70	
**BMI Category**				<0.001
Underweight (<18.5)	1.24	0.14 – 2.35	0.56	
Normal (18.5–24.9)	1.09	0.98 – 1.19	0.05	
Overweight (25–29.9)	1.33	1.25 – 1.42	0.04	
Obese (≥30)	1.84	1.67 – 2.02	0.09	
**Age Group (years)**				<0.001
51–59	1.55	1.36 – 1.73	0.09	
60–69	1.53	1.38 – 1.67	0.07	
70–79	1.37	1.22 – 1.52	0.08	
80+	1.01	0.88 – 1.14	0.07	

Estimates account for the NHANES complex survey design. p-values reflect overall Wald tests for group differences.

Table S4. DHEAS Sensitivity Analysis (NHANES 2021–2023).

Variable	Adjusted OR	95% CI	p-Value
DHEAS (μmol/L)	0.55	0.47–0.63	<0.001
Estradiol	0.98	0.95–1.01	0.168
SHBG	0.99	0.98–0.99	<0.001
Age (years)	0.94	0.90–0.97	<0.01
Non-Hispanic Black	0.79	0.52–1.20	0.256
Non-Hispanic Asian	0.65	0.16–2.60	0.517
Mexican American	1.63	0.77–3.46	0.184
Other Hispanic	0.47	0.24–0.91	<0.05
Other/Multi-Racial	1.98	0.81–4.81	0.123
BMI (kg/m²)	0.96	0.93–1.00	<0.05
Mean Systolic BP	1.01	1.00–1.03	0.141
Mean Diastolic BP	0.98	0.96–1.00	0.118
Sleep Hours	0.89	0.79–0.99	<0.05
PHQ-2 (binary)	0.92	0.42–2.00	0.820

Note: Cycle-restricted survey-weighted logistic regression model including DHEAS. Odds ratios (ORs) represent associations with testosterone

concentrations <30 ng/dL (n = 854). Wald confidence intervals and p-values were derived using survey design–based degrees of freedom (df = 15).

Table S5. Gonadotropin Sensitivity Analysis (NHANES 2021–2023).

Variable	Adjusted OR	95% CI	p-Value
FSH (mIU/mL)	1.01	1.00–1.03	0.086
LH (mIU/mL)	0.96	0.94–0.99	<0.01
DHEAS (μmol/L)	0.54	0.46–0.63	<0.001
Estradiol	0.98	0.95–1.01	0.240
SHBG	0.99	0.98–0.99	<0.001
Age (years)	0.94	0.90–0.98	<0.01
Non-Hispanic Black	0.84	0.52–1.36	0.460
Non-Hispanic Asian	0.6	0.14–2.54	0.460
Mexican American	1.56	0.71–3.45	0.250
Other Hispanic	0.46	0.24–0.90	0.030
Other/Multi-Racial	2.1	0.81–5.42	0.120
BMI (kg/m²)	0.96	0.93–0.99	<0.05
Mean Systolic BP	1.01	0.99–1.02	0.440
Mean Diastolic BP	0.99	0.97–1.01	0.310
Sleep Hours	0.88	0.78–0.99	<0.05
PHQ-2 (binary)	0.91	0.41–2.06	0.820

Cycle-restricted survey-weighted logistic regression model including DHEAS, FSH, and LH. Odds ratios (ORs) represent associations with testosterone concentrations <30 ng/dL (n = 851).

Table S6. Standard Deviation Magnitudes for Continuous Predictors. Standard deviations of continuous variables included in the survey-weighted logistic regression models (NHANES 2011–2016 and 2021–2023).

**Continuous Variable**	**Standard Deviation**
Age (years)	8.94
BMI (kg/m²)	7.15
Systolic BP (mmHg)	19.50
Diastolic BP (mmHg)	12.86
Sleep Hours	1.57
Estradiol (pg/mL)	14.83
SHBG (nmol/L)	38.61

Table S7. Multicollinearity Diagnostics for the Multivariable Model. Generalized variance inflation factors (GVIF) for predictors included in the SD-scaled survey-weighted logistic regression model.

Variable	GVIF	Df	Adjusted GVIF*
Estradiol (per SD)	1.13	1	1.06
SHBG	1.17	1	1.08
Age	1.36	1	1.17
Race/Ethnicity	1.19	4	1.02
BMI	1.23	1	1.11
Mean Systolic BP	1.33	1	1.15
Mean Diastolic BP	1.26	1	1.12
Sleep duration	1.03	1	1.02
PHQ-2 ⩾3	1.04	1	1.02

*Adjusted GVIF is reported as GVIF^(1/(2×Df)) to facilitate comparability across predictors with different degrees of freedom. Values close to 1 indicate minimal collinearity.
